# Sedentary behavior and physical activity in survivors of childhood hodgkin lymphoma: a cross-sectional study

**DOI:** 10.1038/s41598-026-36408-2

**Published:** 2026-01-16

**Authors:** Tomáš Vyhlídal, Jan Dygrýn, Tomáš Kepák, Jarmila Kruseová, František Chmelík

**Affiliations:** 1 Faculty of Physical Culture, Palacký University Olomouc, Olomouc, Czech Republic; 2 International Clinical Research Center, St. Anne’s University Hospital Brno, Brno, Czech Republic; 3 Department of Paediatric Haematology and Oncology, 2nd Medical Faculty, Charles University and University Hospital Motol, Prague, Czech Republic

**Keywords:** Sedentary behavior, Physical activity, Survivors, Hodgkin lymphoma, Accelerometry, Cancer, Health care, Medical research, Oncology

## Abstract

Hodgkin lymphoma (HL) is one of the most common cancers during adolescence. Advances in treatment have achieved survival rates exceeding 90%. However, long-term treatment-related sequelae, including cardiovascular disease and metabolic syndrome, significantly affect the quality of life of survivors. Physical activity (PA) is considered a key strategy to mitigate these risks. Current studies predominantly rely on subjective assessments of movement behavior, which may lack accuracy. This cross-sectional study aims to use device-based monitoring to characterize levels of sedentary behavior and physical activity in survivors of childhood-diagnosed HL. Specific objectives were to evaluate these behaviors across age and gender groups and to assess compliance with physical activity guidelines for the adult population. The study involved 51 participants (59% female), with a median age of 25 years, a median age at diagnosis of 16 years, and a median time since diagnosis of 11 years. PA and sedentary behavior (SB) were measured over seven days using the Axivity AX3 accelerometer with a 24-hour wear protocol. Movement behavior was categorized into SB, light PA (LPA), moderate PA (MPA), and vigorous PA (VPA). Group differences in movement behaviors were examined using non-parametric tests, and results are presented as medians with interquartile ranges. Participants had a median daily time of 704.8 min in SB (IQR 127.9), 181.2 min in LPA (IQR 81.3), 110.2 min in MPA (IQR 68.8), and 2.8 min in VPA (IQR 2.8). Combined moderate-to-vigorous physical activity (MVPA) accounted for a median of 115 min per day (IQR 69.8). Significant differences in LPA were observed: men spent less time in LPA compared to women (*p* = 0.032), and younger participants spent less time in LPA compared to older participants (*p* = 0.006). While 100% of participants met the WHO-recommended threshold of > 150 min of MPA per week, only 14% met the guideline of > 75 min of VPA per week. Our study indicates that survivors diagnosed with childhood HL can achieve the levels of MVPA recommended by current adult PA guidelines, despite undergoing chemotherapy and radiotherapy. Additionally, significant differences in low-intensity PA were identified: men and younger participants spent less time in LPA compared to women and older participants, respectively. These findings highlight the importance of monitoring movement behaviors in long-term follow-up care to identify survivors with insufficient physical activity and excessive sedentary behavior and to implement targeted interventions to reduce long-term cardiovascular and metabolic risk. Given the cross-sectional design, causal relationships and changes in physical activity behavior over time cannot be inferred.

## Introduction

Hodgkin lymphoma (HL) is one of the most frequently diagnosed cancers in adolescence, with the highest incidence observed among individuals aged 15 to 19 years^1^. Globally, it is relatively rare in children, with an estimated incidence of 2–3 cases per 100,000 children per year^2^. In the Czech Republic, the incidence mirrors European trends, ranging between 2.5 and 3 cases per 100,000 children annually^3^. Over the past century, classical HL has transformed from a fatal disease into one of the most treatable malignancies in adolescents, with overall survival rates exceeding 90%^4,5^. Given the globally rising incidence of HL (83,087 new cases in 2020) and the concurrent decline in mortality, the number of long-term survivors is expected to continue increasing^6^. The high survival rate, however, comes at the cost of long-term toxicity from chemotherapy or radiotherapy^5^. Compared to their healthy peers, survivors of childhood HL experience significant emotional and neurocognitive challenges, negatively impacting their quality of life^7^. Survivors also frequently report high levels of fatigue, emotional distress, and cognitive decline, which can lead to reduced physical activity (PA) and increased sedentary behavior (SB)^8^. Additionally, treatment-related effects heighten the risk of metabolic syndrome and cardiovascular diseases, further complicating survivors’ ability to engage in regular PA^9^.

Survivors face elevated risks of secondary malignancies and other chronic health conditions, often leading to higher utilization of healthcare services and hospitalizations, which disrupt their PA routines^10^. Cardiovascular diseases may emerge decades after treatment, underscoring the need for strategies to improve long-term health outcomes^11,12^.

PA plays a crucial role in mitigating the late effects commonly observed in survivors of childhood HL. Regular PA is associated with a lower risk of treatment-related cardiovascular disease and metabolic complications^13–16^ and may help counteract anthracycline- and radiotherapy-induced cardiotoxicity^11,12^. Beyond its cardiometabolic benefits, PA contributes to reduced fatigue, improved psychosocial functioning, and better neurocognitive outcomes in this population^7,8,17^. Despite these advantages, survivors often engage in less PA and accumulate more SB than their healthy peers^8,18–20^. Objective monitoring of PA and SB is therefore important for identifying individuals at elevated risk and for guiding personalized supportive follow-up care^21,22^.

PA offers a promising way to mitigate these risks. Recent evidence highlights its protective effects, including a reduced likelihood of cardiovascular complications associated with HL treatment^13^. Supporting and promoting PA should therefore be a key focus in survivorship care, as it is accessible, effective, and preferred by patients^7,23^.

Despite the benefits of PA, limited research has used device-based monitoring to characterize SB and PA in survivors of childhood HL. Existing studies often rely on subjective self-reports^13,14,17–19^^24^, which may underestimate SB^21^ and overestimate PA^22^.

This study provides a unique contribution by using device-based monitoring to deliver accurate and quantitative assessments of SB and PA in survivors of childhood HL. Furthermore, it examines these parameters a significant time after treatment completion, offering valuable insights into the long-term behavioral patterns of this population.

The primary aim of this cross-sectional study is to characterize SB and PA in survivors of childhood HL using device-based monitoring. Specific objectives include assessing differences across age and gender groups and determining compliance with World Health Organization (WHO) PA guidelines for the adult population.

## Methods

### Study design and participants

This cross-sectional study included 51 participants. Inclusion criteria were: age > 16 years, completion of active oncological treatment for Hodgkin lymphoma during childhood, and a time interval of > 5 years since diagnosis. Exclusion criteria were unrelated disabilities or health disadvantages not associated with previous oncological treatment.

### Recruitment of participants

Participants were recruited in two waves: the first in April 2022 and the second in April 2023. Potential participants were identified through the oncology outpatient registries of the follow-up clinic for childhood cancer survivors at St. Anne’s University Hospital Brno and the Long-Term Follow-up Clinic Motol, Motol University Hospital, Prague. All individuals who met the inclusion criteria were invited by personalized invitation letters. Of the 157 eligible survivors contacted across the two centers, 51 agreed to participate, resulting in a response rate of 32.5%. These dispensary clinics are prominent facilities in the Czech Republic, specializing in preventive and therapeutic care for patients who underwent oncological treatment during childhood and closely collaborating with the only two pediatric comprehensive oncology centers in Brno and Prague.

### Data collection

One month prior to data collection, participants received detailed information about the study, procedures, monitoring device, and evaluation methods. Additional demographic and clinical data, including educational attainment, age, height, weight, diagnosis, disease onset, and treatment type, were collected through questionnaires. Informed consent was required for participation. The study was approved by the Ethics Committee of the Faculty of Physical Culture, Palacký University Olomouc, under reference number 101/2021.

The first data collection took place from May 9–15, 2022, lasting seven days and involving 35 participants. The second wave occurred from May 8–14, 2023, also lasting seven days, with 16 participants. Study materials, monitoring devices, consent forms, and questionnaires were sent to participants’ homes or preferred pick-up locations via a delivery service 2–4 days before the study began.

### Movement behaviors

PA and SB were measured using wrist-worn Axivity AX3 accelerometers (Axivity Ltd., Newcastle, UK) over 7 consecutive 24-hour periods, excluding prolonged activities in water (e.g., swimming and diving). The wrist placement was chosen to maximize participant comfort and adherence to the 24-h, 7-day wear protocol and to ensure comparability with previously published Axivity AX3 protocols and cut-points based on wrist-worn raw acceleration data. The accelerometers were set to collect raw acceleration data at 100 Hz on all three axes. Device initialization and data downloading were performed using Open Movement software (Open Lab, Newcastle University, UK). The collected .cwa files were analyzed using the open-source R package GGIR (v3.1-2, https://cran.r-project.org/web/packages/GGIR/) in accordance with the standardized protocols described by Migueles et al.^25^. Appropriate acceleration thresholds were applied to identify SB and PA intensities: <46 milligravity units (m*g*) for SB, 46–92.9 m*g* for LPA, 93–417.9 m*g* for MPA, and ≥ 418 m*g* for VPA^26,27^. A valid day was defined as one with ≥ 75% wear time during wake periods and ≥ 75% wear time during sleep periods. Participants were included in the analysis only if they had valid data for all 7 days. All 51 participants who agreed to accelerometer monitoring provided valid data for all 7 days. The median device wear time was 23.88 h per day (IQR 0.34).

### Statistical analysis

Statistical analyses were performed using IBM SPSS Statistics software, version 25 (IBM SPSS, Inc., Chicago, IL, USA). Descriptive statistics, including medians and interquartile ranges (IQR), were calculated to summarize the sample characteristics. Nonparametric Mann-Whitney U tests were used for group comparisons based on sex and age group. A significance level of α = 0.05 was used. Effect sizes were interpreted as follows: 0.2 ≤ d < 0.5 (small), 0.5 ≤ d < 0.8 (medium), and d ≥ 0.8 (large).

## Results

### Sample characteristics

Table 1 summarizes the descriptive characteristics of the study sample. The median time since diagnosis was 11 years (10 years for men and 13 years for women). The sample was divided by gender (men and women) and age groups (< 25 years and ≥ 25 years), with the latter grouping reflecting the completion of early adulthood, which is characterized by peak physical fitness^28^.

**Table 1:** Descriptive characteristics of the study Sample.

Group	*N*	Age (years)	Age at Diagnosis (years)	Time Since Treatment (years)	BMI (kg/m²)
Men	21	22 (10)	15 (7)	10 (7.5)	22.84 (7.5)
Women	30	28 (14)	16 (3)	13 (13.75)	23.85 (8.4)
< 25 years	24	21.5 (4)	14 (6)	7 (4.75)	22.98 (7.5)
≥ 25 years	27	33 (14)	16 (2)	17 (15)	23.90 (6.9)
Total	51	25 (12)	16 (3)	11 (10)	23.80 (7.8)

Note. All values are presented as median (IQR).

Table 2 presents the distribution of key demographic and clinical characteristics of the study sample, including gender, type of diagnosis, treatment methods, educational attainment, smoking status, and BMI categories. A higher proportion of women (58.8%) compared to men (41.2%) participated in the study. The majority of participants (86.3%) underwent radiotherapy in addition to chemotherapy, while only 7.8% received a stem cell transplant. Regarding educational attainment, most survivors completed secondary education (56.9%), followed by university education (29.4%), and primary school (13.7%). Smoking was rare among participants, with only 3.9% identified as smokers.

**Table 2:** Percentage distribution of sample characteristics.

Variable	Category	*N*	%
Gender	Male	21	41.2
	Female	30	58.8
Diagnosis	C810	3	5.9
	C811	27	52.9
	C812	14	27.5
	C819	7	13.7
Chemotherapy	Yes	51	100
	No	0	0
Radiotherapy	Yes	44	86.3
	No	7	13.7
Stem Cell Transplant	Yes	4	7.8
	No	47	92.2
Education	Primary school	7	13.7
	Secondary school	29	56.9
	University	15	29.4
Smoking	Yes	2	3.9
	No	49	96.1
BMI	< 18.5	1	2.0
	18.5–24.9	30	58.8
	25–29.9	13	25.5
	> 30	7	13.7

### Physical activity and sedentary behavior

Participants demonstrated varying levels of movement behavior, with notable differences in light physical activity (LPA) between genders and age groups (Table 3). Men spent significantly less time in LPA compared to women (median 151.8 min/day vs. 202.1 min/day, *p* = 0.032, d = 0.6). Similarly, younger participants (< 25 years) spent significantly less time in LPA than older participants (≥ 25 years) (median 150.8 min/day vs. 306.8 min/day, *p* = 0.006, d = 0.77). No significant differences were found for sedentary behavior (SB), moderate physical activity (MPA), vigorous physical activity (VPA), or combined moderate-to-vigorous physical activity (MVPA) between genders or age groups.

**Table 3:** Sedentary behavior and physical activity Characteristics.

Movement Behavior	Total (*N* = 51)	Men (*N* = 21)	Women (*N* = 30)	*p*-value	d	< 25 years (*N* = 24)	≥ 25 years (*N* = 27)	*p*-value	d
SB (min/day)	704.8 (127.9)	712.9 (88.2)	687.0 (158.5)	0.411	0.23	715.9 (130.6)	693.4 (144.4)	0.282	0.30
LPA (min/day)	181.2 (81.3)	151.8 (63.8)	202.1 (80.7)	**0.032**	**0.60**	150.8 (58.3)	306.8 (63.6)	**0.006**	**0.77**
MPA (min/day)	110.2 (68.8)	104.5 (47.6)	113.6 (93.5)	0.251	0.32	111.3 (54.3)	106.2 (71.6)	0.910	0.03
VPA (min/day)	2.8 (4.2)	3.9 (7.1)	2.1 (3.8)	0.085	0.48	3.0 (3.6)	1.7 (4.7)	0.406	0.23
MVPA (min/day)	114.7 (69.8)	108.0 (51.4)	117.4 (100.0)	0.221	0.34	114.7 (54.6)	114.7 (74.1)	0.940	0.02

Note. Movement behavior durations are presented as median (IQR), d: Effect size coefficient, SB: Sedentary behavior, LPA: Light physical activity, MPA: Moderate physical activity, VPA: Vigorous physical activity, MVPA: Moderate-to-vigorous physical activity.

All participants met the WHO recommendation of > 150 min of MPA per week, as shown in Table 4. Compliance with the more demanding guideline of > 75 min of VPA per week was low overall, with 14% of participants achieving this target. While there were observed differences in compliance between genders (24% of men vs. 7% of women) and age groups (15% of older participants vs. 13% of younger participants), these differences were not statistically significant based on the chi-squared test (gender: χ²(1) = 2.91, *p* = 0.088; age: χ²(1) = 0.04, *p* = 0.839).

**Table 4:** Compliance with physical activity recommendations by gender and age Group.

Physical Activity Recommendation	Total	Men	Women	< 25 years	≥ 25 years
> 150 min MPA/week	51 (100%)	21 (100%)	30 (100%)	24 (100%)	27 (100%)
> 75 min VPA/week	7 (14%)	5 (24%)	2 (7%)	3 (13%)	4 (15%)

Note. MPA: Moderate physical activity, VPA: Vigorous physical activity. Statistical comparisons of compliance rates were performed using chi-squared tests. None of the observed differences reached statistical significance (*p* > 0.05).

## Discussion

This study provides new insights into the movement behavior of survivors of childhood HL. Participants demonstrated high levels of SB and notable differences in LPA across gender and age groups, with men and younger participants engaging in less LPA. While adherence to MPA recommendations was universal, compliance with VPA guidelines was limited. These findings emphasize the need to address specific behavioral patterns in this population to mitigate long-term health risks.

From a clinical perspective, the movement behavior patterns observed in our cohort are concerning. High sedentary time has been associated with increased cardiovascular and cerebrovascular morbidity, metabolic complications and poorer health-related quality of life in cancer survivors and in the general population^20,29,30^. Survivors of childhood HL are already at elevated risk of treatment-related cardiovascular disease and other late effects, and unfavorable movement behaviors may further exacerbate this risk^9,11–14^. Although we did not assess clinical outcomes such as cardiovascular events, neurocognitive function or patient-reported quality of life, our findings highlight modifiable behavioral targets that could be addressed within survivorship care. Longitudinal and interventional studies are needed to determine whether changing sedentary behavior and physical activity patterns in this population translates into measurable improvements in these clinical outcomes.

A sedentary lifestyle is a significant risk factor associated with cardiovascular disease^15,29^. According to the American Heart Association (AHA), SB is one of the leading preventable causes of mortality, with an inverse linear relationship between physical activity levels and overall mortality. Epidemiological evidence suggests that prolonged sedentary time, particularly exceeding 10 h daily, substantially increases cardiovascular risk^30^. In this study, SB exceeded recommended thresholds, a finding consistent with previous research reporting high SB levels among young cancer survivors, including those with HL^20^. While men and younger participants appeared to spend slightly more time in SB compared to women and older participants, these differences were not statistically significant and warrant further exploration in larger cohorts.

Differences in LPA were more pronounced, with women and older participants engaging in significantly more LPA. This aligns with prior studies suggesting that women may prefer lower-intensity physical activities^31,32^. The higher LPA levels in older participants, however, were unexpected. This could reflect lifestyle factors such as shifts in work routines, increased prioritization of health, or other influences, and future studies should investigate these underlying mechanisms.

Although current PA guidelines primarily emphasize achieving sufficient MVPA, they also explicitly recommend limiting sedentary time and replacing it with PA of any intensity, including LPA. According to the WHO, adults with chronic conditions should minimize the amount of time spent sedentary, and replacing sedentary time with PA of any intensity provides health benefits^33^. For survivors of childhood HL, who are exposed to treatment-related cardiovascular and metabolic risk, increasing LPA throughout the day may therefore represent a realistic and clinically meaningful strategy to reduce prolonged SB and support cardiometabolic health, even for those who already meet MVPA targets but still accumulate large amounts of sedentary time. In this context, the observed differences in LPA between survivor subgroups in our study may help identify individuals who rely more heavily on SB and who might benefit from follow-up strategies that focus not only on maintaining sufficient MVPA but also on incorporating more light-intensity movement into everyday routines.

In our cohort, all survivors accumulated at least 150 min of MPA per week, resulting in a 100% compliance rate with current adult PA guidelines when based on accelerometer-derived MPA. This is particularly encouraging given the known benefits of MPA in reducing cardiovascular and metabolic risks^31^. At first sight, this proportion appears unusually high compared with many self-report–based studies in both cancer survivors and the general population. However, current PA recommendations were largely developed from questionnaire-based data and historically focused on MVPA accumulated in bouts of at least 10 min, whereas in the present study we used wrist-worn Axivity AX3 accelerometers and analyzed raw triaxial data in 5-second epochs. This approach tends to yield substantially higher estimates of weekly MPA than self-report, and accelerometer-derived minutes of MPA may therefore not be directly compatible with guideline thresholds. Ramakrishnan et al., who used the same device and identical threshold in more than 90,000 adults from the United Kingdom, also reported very high levels of MPA (743.2 min per week), far exceeding the recommended 150 min per week^34^. The similarity between our findings and those of Ramakrishnan et al. suggests that the apparently high guideline compliance in our cohort is at least partly driven by the methodology used to derive MVPA from wrist accelerometry rather than by uniquely high activity levels in our sample.

Methodological influences on guideline fulfilment are further illustrated by Ekblom-Bak et al., who showed that the proportion of adults meeting PA recommendations varied dramatically depending on the accelerometer metrics and definitions applied, ranging from very low to very high fulfilment rates^35^. These observations underline that estimates of guideline compliance based on accelerometry are highly sensitive to analytical decisions (e.g. axis, epoch length, bout definition and intensity cut-points), and that comparisons across studies – and with questionnaire-based recommendations – are therefore limited. Our findings should thus be interpreted as indicating that survivors regularly engage in activities that reach the intensity range classified as MPA by current wrist-based cut-points, rather than as definitive evidence that all survivors meet PA recommendations in the way originally operationalized in self-report research. In contrast, compliance with VPA guidelines was notably lower, which is consistent with broader trends observed in both the general population and cancer survivor cohorts^20,36^. This pattern highlights higher-intensity activity as a potential target for interventions to further improve the cardiometabolic risk profile of survivors of childhood HL.

The study’s strengths include its use of device-based monitoring, which ensures accurate and objective assessments of PA and SB. This approach minimizes biases often associated with self-reported measures and provides robust data on movement patterns. Additionally, the significant time elapsed since treatment completion offers a valuable perspective on long-term health behaviors among HL survivors.

However, a potential limitation of this study is selection bias. The study may have disproportionately attracted participants who are more motivated to maintain a healthy lifestyle or engage in regular follow-up care. These individuals may be more likely to exhibit better adherence to PA guidelines and may not fully represent the broader population of HL survivors, particularly those with lower levels of activity or inconsistent follow-up attendance. Consequently, the findings may primarily reflect the outcomes of long-term survivors with good adherence to treatment and follow-up care. Future research should explore strategies to include less active or less engaged survivors to provide a more comprehensive understanding of physical activity and sedentary behavior patterns in this population.

Second, the sample size of our cohort was relatively small, which reduces the precision of the estimates and limits the statistical power, particularly for subgroup comparisons. We were not able to adjust for potentially important covariates such as treatment modality, treatment intensity, time since diagnosis or comorbidities, which may influence movement behaviors. Therefore, the subgroup analyses should be considered exploratory, are not directly generalizable to all survivors of childhood HL, and their results should be interpreted with caution. At the same time, childhood HL is a rare disease, and our study includes a substantial proportion of survivors from a single national cohort, providing valuable preliminary data that warrant confirmation in larger, ideally multicenter studies.

Third, we did not assess psychosocial determinants of movement behaviors, such as cancer-related fatigue, motivation, psychological distress or social support. These factors are highly relevant in cancer survivorship and are likely to influence survivors’ ability and willingness to engage in PA, and they may partly explain the variability in sedentary time and PA levels observed in our cohort. Future research should therefore combine device-based measurements of SB and PA with validated assessments of psychosocial constructs to better understand the mechanisms underlying physical inactivity and to inform tailored interventions for survivors of childhood HL.

## Conclusions

Our study suggests that survivors of childhood HL can, despite undergoing intensive treatments such as chemotherapy and radiotherapy, achieve the levels of MVPA recommended by current adult PA guidelines. Additionally, the study found significant differences in low-intensity physical activity across gender and age groups.

Monitoring and assessing current levels of sedentary behavior and physical activity is essential for healthcare providers, as physical activity directly influences long-term health and the risk of late treatment effects in survivors of childhood Hodgkin lymphoma. This approach enables the identification of patients with insufficient activity levels and the development of targeted interventions to mitigate the risk of cardiovascular and metabolic complications. These insights also support the evaluation of preventive strategies and facilitate personalized care. Considering the treatment-related toxicity and potential cardiovascular risks as late effects of therapy, evaluating sedentary behavior and physical activity should become a key component of long-term follow-up care for HL survivors.

## Data Availability

The datasets generated during and/or analysed during the current study are available from the corresponding author on reasonable request.
